# Diabetic retinopathy as the primary predictor of mild cognitive impairment in type 2 diabetes: Insights from machine learning models

**DOI:** 10.1371/journal.pone.0332442

**Published:** 2025-09-26

**Authors:** Fatima Zahra Rhmari Tlemçani, Faiçal Aitlahbib, Soukaina Laidi, Najib Alidrissi, Jehanne Aasfara, Saloua Elamari, Asma Chadli, Imane Motaib

**Affiliations:** 1 Faculty of Medicine, Laboratory of genomics, Epigenetics, Personalized and Predictive Medicine, Mohammed VI University of Health Sciences (UM6SS), Casablanca, Morocco; 2 Innovx, Mohammed VI Polytechnic University, Benguerir, Morocco; 3 Department of Endocrinology Diabetology Metabolic Disease and Nutrition, Faculty of Medicine, Cheikh Khalifa International University Hospital, Mohammed VI University of Health Sciences (UM6SS), Casablanca, Morocco; 4 Faculty of Medical Sciences, UM6P Hospitals, Mohammed VI Polytechnic University, Benguerir, Morocco; Ferdowsi University of Mashhad, IRAN, ISLAMIC REPUBLIC OF

## Abstract

Mild cognitive impairment (MCI) is a significant and increasingly recognized problem in individuals with type 2 diabetes mellitus (T2DM). This study aims to develop a machine-learning model to predict MCI in patients with T2DMThe dataset was obtained from a prospective cohort conducted at the Sheikh Khalifa Ibn Zaid Hospital, Casablanca, Morocco, and was randomly split into three parts. Machine learning models were trained using the training dataset, and their performance was assessed on the test dataset. The unseen data was reserved for final validation. Subsequently, recursive feature elimination was applied with the top two algorithms to identify and retain the most impactful features for predicting MCI. Then, we retrained the models using the selected variables. Finally, the variables most contributing to the prediction of MCI were represented in a Shapley Additive Explanations SHAPE value plot to better understand their contribution and their ranking in MCI prediction.The dataset included 100 patients. Extra Trees classifier was the best-performing model for mild cognitive impairment (Accuracy: 0·9310/AUC: 0·9667/ Recall: 0·9333/ Precision: 0·9333/F1: 0·9333). The most contributing factors to MCI in patients with type 2 diabetes were, respectively, diabetic retinopathy, age, serum LDL cholesterol level, microalbuminuria, HbA1c, and, serum creatinine level. Our findings suggest avenues for early intervention that could prevent the progression of cognitive impairment among patients with type 2 diabetes.

## Introduction

Mild cognitive impairment (MCI) is a cognitive dysfunction defined as a symptomatic pre-dementia state that can either develop into dementia, remain stable, or regress [1]. Its prevalence ranges from 10% to 20% among adults over 65 years old and increases with age [2]. In individuals with type 2 diabetes mellitus (T2DM), MCI is a significant and increasingly recognized problem. Studies have shown that individuals with type 2 diabetes have a prevalence of MCI of approximately 45% [3]. Additionally, studies have suggested that the incidence of MCI is higher among patients with type 2 diabetes compared to those without diabetes [4].

This cognitive impairment can affect daily activities, including mental agility, focus, perception, knowledge acquisition, recall, and the ability to perform executive functions [5]. These disorders can significantly impact diabetes management, which relies on patient involvement in lifestyle management and treatment compliance.

Several factors have been proposed to contribute to the development of MCI in patients with type 2 diabetes, including insulin resistance, poor glycemic control, glycemic fluctuations, degenerative complications, and diabetes duration [4]. However, identifying these factors is challenging, and their definition would enable personalized and early management of patients with type 2 diabetes and MCI.

Recent advances in machine learning have paved the way for predictive analytics to identify key risk factors for diabetes and its complications [6,7], providing new insights into personalized healthcare approaches. However, there is a significant gap in the literature on the use of machine learning techniques for detecting and predicting MCI in this population. Only a few studies have explored the application of machine learning in this context.

The aim of this study is to develop a machine-learning model for predicting mild cognitive impairment in patients with type 2 diabetes.

## Materials and methods

### Data source

The dataset was obtained from a prospective cohort conducted at the Sheikh Khalifa Ibn Zaid Hospital, Casablanca, Morocco. The study aimed to assess the prevalence and risk factors for mild cognitive impairment in patients with type 2 diabetes. A total of 100 patients were recruited between May and September 2021. For this study, we included patients with type 2 diabetes mellitus, aged between 40 and 75 years, with a duration of diabetes exceeding five years. Patients with a history of ischemic stroke or depression, those taking antiepileptic drugs, those with glycosylated hemoglobin (HbA1c) levels below 4%, or those aged over 75 years were excluded.

The primary outcome being predicted in this study is MCI, assessed using the Mini-Mental State Examination (MMSE), a widely used cognitive screening tool. All assessors had extensive experience in managing patients with T2DM and were trained in administering and interpreting the MMSE. A detailed description of the methodology and study design is provided in our previous manuscript [8].

As all patients were recruited from this single site, there were no variations in model parameter values and performance due to differences in hospitals or countries. Therefore, handling and quantifying heterogeneity across clusters was not applicable in this study.

### Exploratory data analysis (EDA) and variable selection

The study size was determined by including all patients who met the inclusion criteria during the study period, ensuring comprehensive data collection. The dataset comprises 21 variables and 100 records. The target parameter “MCI” is balanced (47%/53%). The histograms representing the distributions of the other variables are shown in S1 Fig. To enhance model discrimination, we assessed the strength of linear correlation between the variables in the dataset using Pearson correlation. This step enables the selection of variables most associated with mild cognitive impairment. No variable showed a significant linear correlation with the target parameter “MCI”. The correlation coefficient values are presented in S2 Fig. This finding rules out the possibility of using a simplified model with a representative small cluster of variables. Therefore, the 21 variables were included in nonlinear machine learning methods.

### Data preprocessing

Since there were no missing values in the dataset, we did not run an imputation procedure. To improve the predictive power of our algorithms, we first addressed categorical variables with more than two possible values, using the one-hot encoding technique. In addition, to ensure fair treatment of all variables and to prevent any single feature from dominating the model, we normalized the dataset using the Z-score method.

### Data splitting and metrics

The data was randomly divided into two parts. The first part, which represented 95% of the data, was then split into two sections: 70% was used as the training set and 30% as the test set. Machine learning models were trained using the training dataset, and their performance was assessed on the test dataset. The remaining 5% of the total data, referred to as the ‘unseen data’, was reserved for final validation. After selecting the best model, it was validated on the test dataset and then finalized on the unseen data. To ensure consistency in the healthcare environment and patient population, all data used for both the development and evaluation phases were obtained from the same healthcare setting, Cheikh Khalifa Ibn Zaid Hospital. For model optimization, we adjusted the hyperparameters using the k-fold cross-validation approach. We did not perform any specific recalibration or updating of the model parameters. We evaluated the effectiveness of different machine learning models using specific performance metrics, including the confusion matrix, accuracy, area under the curve (AUC), recall, precision, and, F1 score.

### Supervised machine learning models and feature-selection techniques

We initially utilized a variety of widely recognized supervised machine learning algorithms, including CatBoost, Decision Tree, Gradient Boosting, Random Forest, Extra Trees, Extreme Gradient Boosting, Light Gradient Boosting Machine, Naive Bayes, K Neighbors Classifier, Linear Discriminant Analysis, Ridge Classifier and SVM-linear kernel, Logistic Regression, Quadratic Discriminant Analysis and, Dummy Classifier. Initially, all dataset features were used to train the models because there was no linear correlation between the target parameter ‘mild cognitive impairment’ and other variables. We conducted a fine-tuning approach using ‘random grid search’ and ‘Optuna random search’ to optimize the models’ hyperparameters and enhance predictive metrics. Subsequently, we applied Recursive Feature Elimination (RFE) with the top two algorithms to identify and retain the most impactful features for predicting MCI. The models were then retrained using the selected variables to enhance accuracy and minimize overfitting risks. The most contributing variables for predicting MCI using the best-performing model were represented in a Shapley Additive Explanations SHAPE value plot to better understand their contribution and ranking in MCI prediction.

### Statistical analysis

All statistical analysis was performed using Pycaret Auto ML library version 2.0 on Python version 3.6.

### Ethical considerations

The study was approved by the Ethics Committee of Mohammed VI University of Health Sciences (approval number: CERB/UM6SS/11/21). Data were actively collected from participants between 1^st^ May and 30^th^ September 2021 at Cheikh Khalifa Ibn Zaid Hospital, Casablanca, Morocco. Due to the non-invasive nature of the study, all participants provided oral informed consent before enrollment in the study. The research team ensured that each participant received a detailed explanation of the study’s objectives, procedures, potential risks, and benefits. To comply with ethical standards, an investigator documented the verbal consent in the study records. The dataset was originally collected for a previously published descriptive study and later used for the development of machine learning models to evaluate predictors of mild cognitive impairment (MCI). No personally identifiable information was collected, and all data were fully anonymized prior to analysis. Patient confidentiality was maintained following institutional and ethical guidelines.

## Results

### Patient characteristics

The study included 100 patients with a median age of 65 years (interquartile range [IQR]: 59−70 years), 65% of whom were male and 35% were female. The prevalence of MCI did not differ by gender (female 17/35, 48.6%; male 35/65, 53.8%; χ^2^ = 0.254, p = 0.615). Gender-stratified analyses (crude and multivariable with gender forced in) showed no independent association with MCI (male vs female: crude OR 1.24 [95% CI 0.54–2.81]; adjusted OR 0.81 [95% CI 0.28–2.36]). The median BMI was 26·5 kg/m^2^ (IQR: 23−30 kg/m^2^). The median duration of diabetes was 15 years (IQR: 9−20 years), and the median HbA1c was 8% (IQR: 7·1%−9·13%). The prevalence of MCI was 47·5%. A comprehensive description of the study population is provided in Table 1.

**Table 1:** Descriptive characteristics of the study population (n = 100).

Variable	Value
Age, median (IQR), years	65 (59–70)
Male, n (%)	65 (65%)
Female, n(%)	35(35%)
Duration of diabetes, median (IQR), years	15 (9–20)
Tobacco use, n (%)	18 (18%)
Hypertension, n (%)	72 (72%)
Dyslipidemia, n (%)	53 (53%)
BMI, median (IQR), kg/m²	26.5 (23–30)
Hyperuricemia, n (%)	9 (9%)
Diabetic retinopathy, n (%)	39 (39%)
Diabetic nephropathy, n (%)	17 (17%)
Peripheral neuropathy, n (%)	50 (50%)
Coronary artery disease, n (%)	27 (27%)
Peripheral artery disease, n (%)	30 (30%)
Podiatric grade 0, n (%)	64 (64%)
Podiatric grade 1, n (%)	14 (14%)
Podiatric grade 2, n (%)	10 (10%)
Podiatric grade 3, n (%)	12 (12%)
Amputation, n (%)	6 (6%)
HbA1c, median (IQR), %	8 (7.1–9.13)
LDL cholesterol, median (IQR), g/L	0.90 (0.71–1.20)
Positive microalbuminuria, n (%)	17 (17%)
Creatinine, median (IQR), mg/L	7.25 (6.3–9.93)
Uric acid, median (IQR), mg/L	53 (36.5–63)

This table is adapted from Rhmari Tlemçani FZ et al. (2022), “Factors Associated With Mild Cognitive Impairment in Patients With Type 2 Diabetes: A Cohort Study”, Cureus 14(8):e28305, under the terms of the Creative Commons Attribution License CC BY 4.0 [8].

### Comparison of models for predicting MCI

Using the training dataset, we compared the performance of the considered predictive models.

Table 2 shows a comparison of the performance of predictive models using the training dataset. The Catboost classifier achieved the best performance (Accuracy: 0·8024/AUC:0·8639/Recall: 0·7833/Precision: 0·85835/F1: 0·7912) followed by the Decision tree classifier (Accuracy: 0·8000/AUC:0·8000/ Recall: 0·8583/Precision: 0·7967/F1: 0·8210) and the Gradient Boosting Classifier (Accuracy: 0·8000/AUC: 0·7972/ Recall: 0·7417/Precision: 0·8750/F1: 0·7931).

**Table 2:** Comparison of the performance of the predictive models using the training dataset.

Machine learning Model	Accuracy	AUC	Recall	Precision	F1
CatBoost Classifier^a^	0·8024	0·8639	0·7833	0·8583	0·7912
Decision Tree Classifier	0·8000	0·8000	0·8583	0·7967	0·8210
Gradient Boosting Classifier	0·8000	0·7972	0·7417	0·8750	0·7931
Random Forest Classifier^b^	0·7857	0·7694	0·7083	0·8750	0·7612
Extra Trees Classifier^a^	0·7738	0·7667	0·6750	0·8600	0·7278
Extreme Gradient Boosting	0·7405	0·7583	0·6333	0·8667	0·7219
Light Gradient Boosting Machine	0·7286	0·7972	0·6333	0·8333	0·7124
Naive Bayes	0·7095	0·6472	0·5333	0·8833	0·6381
K Neighbors Classifier	0·6857	0·6917	0·6167	0·7000	0·6312
Linear Discriminant Analysis	0·6762	0·6250	0·6500	0·7233	0·6601
Ridge Classifier	0·6619	0·0000	0·6167	0·7067	0·6363
SVM – Linear Kernel	0·6310	0·0000	0·6417	0·6717	0·6379
Logistic Regression	0·6190	0·6333	0·5833	0·6433	0·5947
Quadratic Discriminant Analysis	0·5286	0·5083	0·3500	0·2714	0·2848
Dummy Classifier	0·5286	0·5000	1·0000	0·5286	0·6903

^a^Fine-tuned model using Optuna random search (Optuna library).

^b^Fine-tuned model using random grid search (sikit-learn library)

In the following step, we selected the best-performing models from the training dataset and evaluated them using the test set. The Extra Trees Classifier was the best-performing model (Accuracy:0·8276/AUC:0·8619/Recall: 0·8000/ Precision: 0·8571/F1: 0·8276) followed by the Gradient Boosting Classifier (accuracy:0·7586/AUC:0·8619/ Recall: 0·8000/ Precision: 0·7500/F1: 0·7742) and the Extreme Gradient Boosting (Accuracy:0·7241/AUC:0·8619/ Recall: 0·7333/ Precision: 0·7333/F1: 0·7333). Table 3 shows additional models and metrics.

**Table 3:** Comparison of the selected predictive models’ performance using the training test set.

Machine Learning Model	Accuracy	AUC	Recall	Precision	F1
Extra Trees Classifier^a^	0·8276	0·8619	0·8000	0·8571	0·8276
Gradient Boosting Classifier	0·7586	0·8619	0·8000	0·7500	0·7742
Extreme Gradient Boosting	0·7241	0·8619	0·7333	0·7333	0·7333
Random Forest Classifier^b^	0·6897	0·8810	0·6000	0·7500	0·6667
Decision Tree Classifier	0·6552	0·6524	0·7333	0·6471	0·6875
CatBoost Classifier^a^	0·5862	0·7333	0·4000	0·6667	0·5000

^a^Fine-tuned model using Optuna random search (Optuna library).

^b^Fine-tuned model using random grid search (sikit-learn library).

### Variable selection

The Recursive Feature Elimination (RFE) method was used to select features for the top two models (Extra Trees Classifier and Gradient Boosting Classifier) for predicting MCI in patients with type 2 diabetes. The most significant variables for predicting MCI were age, diabetic retinopathy, microalbuminuria, serum LDL-cholesterol, serum creatinine, and, glycated hemoglobin (HbA1c).

### Machine learning models comparison after variables selection

**Fig 1:**
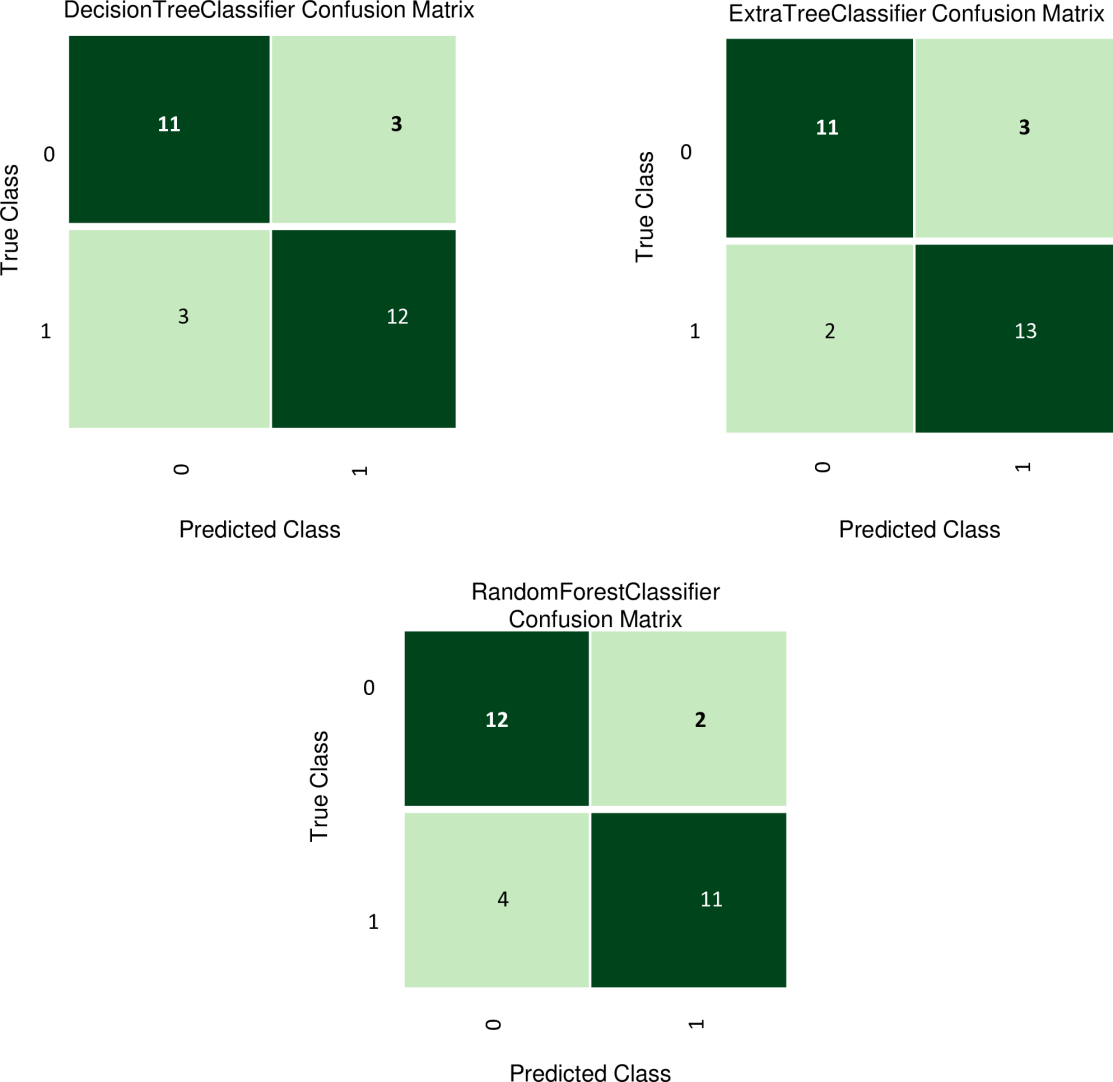
Confusion matrix of the top 3 models.

The selected variables were used to compare the supervised machine learning models once again to determine the best-performing models, as shown in S1 Table. A shortlist of models with an accuracy higher than 0·8 was then chosen to validate our models with the test dataset. Table 4 shows the top 3 best-performing models for MCI prediction using the test set. These models are the Extra Trees Classifier (Accuracy: 0·8276/AUC: 0·8952/Recall: 0·8667/Precision: 0·8125/F1: 0·8387), the Decision Tree Classifier (Accuracy: 0·7931/AUC: 0·7929/Recall: 0·8000/Precision: 0·8000/F1: 0·8000) and, the Random Forest Classifier (Accuracy: 0·7931/AUC: 0·9119/Recall: 0·7333/Precision: 0·8462/F1: 0·7857). The confusion matrix of the top 3 models is represented in Fig 1.

**Table 4:** Comparison of the performance of the predictive models using on test validation with feature selection and short listing of models with accuracy higher than 0·8.

Machine learning Model	Accuracy	AUC	Recall	Precision	F1
Extra Trees Classifier^a^	0·8276	0·8952	0·8667	0·8125	0·8387
Decision Tree Classifier	0·7931	0·7929	0·8000	0·8000	0·8000
Random Forest Classifier^b^	0·7931	0·9119	0·7333	0·8462	0·7857
CatBoost Classifier^b^	0·6897	0·8857	0·6000	0·7500	0·6667
Gradient Boosting Classifier^b^	0·6552	0·8714	0·4667	0·7778	0·5833

^a^Fine-tuned model using Optuna random search (Optuna library).

^b^Fine-tuned model using random grid search (sikit-learn library).

**Fig 2:**
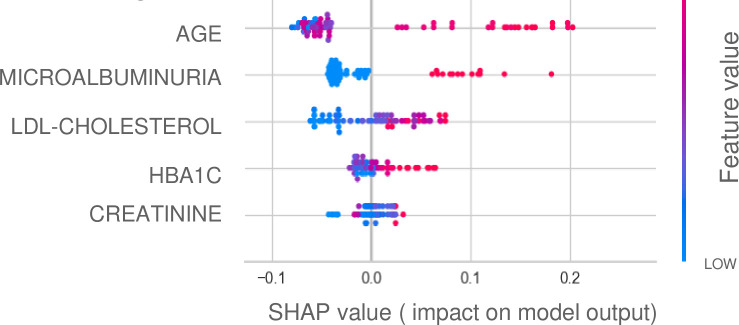
SHAPE value plot for extra trees classifier model.

As a final step, we used the unseen dataset with the validated trained model to predict MCI, using the best performing model, Extra Trees Classifier. This model achieved an accuracy of 0·9310, an AUC of 0·9667, a recall of 0·9333, a precision of 0·9333, and, an F1 score of 0·9333. Additionally, we visualized the impact of different predictors using a Shapley Additive Explanations (SHAP) value plot derived from the Extra Trees Classifier model. Fig 2 shows the factors that contribute the most to MCI in patients with diabetes, represented in a SHAPE value plot. Diabetic retinopathy was the predominant factor, with an increased risk of MCI in patients diagnosed with this condition compared to those without it. The second parameter, age, was found to be associated with a higher risk of MCI. However, there is variability among patients that can result in a lower predicted risk. The third factor identified was microalbuminuria. Patients with elevated levels of microalbuminuria are at a greater risk of developing MCI compared to those without microalbuminuria. Additionally, the SHAP value plot identified serum LDL cholesterol levels as the fourth parameter. The plot suggests that individuals with the lowest serum LDL cholesterol levels may have a reduced risk of MCI onset. Conversely, elevated serum LDL cholesterol levels were associated with an increased risk of MCI, although the relationship appeared more variable than for previous factors.

The fifth factor was HbA1c, which has a variable impact on the prediction of MCI. Generally, elevated HbA1c levels were associated with an increased risk of MCI, while not all instances of lower HbA1c contributed to a reduced risk. The last contributing parameter to MCI was serum creatinine levels. The analysis of SHAP values for creatinine indicates that higher levels of creatinine are associated with an increased risk of MCI, while lower levels are associated with a decreased risk.

## Discussion

Mild cognitive impairment is recognized as a comorbidity associated with diabetes mellitus. Several studies have shown a higher prevalence of MCI among patients with type 2 diabetes [9,10]. In our study, the prevalence of MCI was 47·5%. Moreover, patients with type 2 diabetes are more likely to have a poorer prognosis of MCI, with a 1.7-fold increased risk of progression to dementia [11]. These findings highlight the importance of identifying risk factors for MCI in patients with type 2 diabetes, to prevent its onset and progression. Recently, Jiang et al. developed and validated a clinical nomogram based on logistic regression to predict MCI in elderly patients with type 2 diabetes, emphasizing the relevance of individualized prediction tools in this population [12].

In this context, several studies have explored the use of machine learning in classifying, screening, and predicting the progression of mild cognitive impairment (MCI) in the general population [13–15]. However, only a few published studies have investigated the use of machine learning for detecting or predicting MCI among patients with type 2 diabetes. In one study, the authors developed an MRI-based machine learning model to detect MCI in patients with type 2 diabetes [16]. In the second study, machine learning algorithms were used to assess cognitive impairment more broadly, without specific emphasis on MCI among patients with diabetes [17]. More recently, Maimaitituerxun et al. employed a decision tree algorithm on a cohort of 1,001 patients with type 2 diabetes, identifying age, educational level, income, physical activity, and diabetic complications, including retinopathy, as relevant predictors of MCI [18]. However, their study relied on a single-model approach and did not systematically compare algorithm performance or explore advanced feature selection methods.

Our study demonstrates that machine learning models can accurately predict mild cognitive impairment in patients with type 2 diabetes using machine learning algorithms. The Extra Trees Classifier emerged as the best-performing model, achieving high scores across multiple evaluation metrics: Accuracy (0·9310), Area Under the Curve (AUC) (0·9667), Recall (0·9333), Precision (0·9333), and F1 Score (0·9333). These results indicate that the model is highly effective in predicting MCI with high accuracy and a strong balance between precision and recall, minimizing both false positives and false negatives.

Additionally, Recursive Feature Elimination (RFE) was used to identify the variables most strongly associated with Mild Cognitive Impairment (MCI) among patients with type 2 diabetes. We found that diabetic retinopathy (DR) was the most significant contributing factor to MCI. This result is consistent with the findings of a meta-analysis of 10 studies that showed a significant association between DR and cognitive impairment, as reflected by an odds ratio of 2·24 (95% CI, 1·89–2·66; I^2^ = 0·8%) [19]. This association highlights the intricate relationship between diabetic retinopathy and cognitive dysfunction. Both conditions share pathophysiological phenomena related to microvascular damage in the retina and brain caused by chronic hyperglycemia-induced oxidative stress and inflammation [20]. Cheung et al. also demonstrated the contribution of artificial intelligence in studying the relationship between the retina and cognitive impairment by developing a deep learning model to screen for Alzheimer’s disease using retinal photography [21]. Age has been identified as the second contributing factor to MCI, particularly in older patients with type 2 diabetes. This finding is consistent with existing research that highlights age as a critical risk factor for MCI in patients with type 2 diabetes. Studies have shown that the rate of cognitive decline in individuals with T2DM is 1·5 to two times faster than in those without this condition [4]. We also found a lower predicted risk among some older patients. This variability may be attributed to the interaction with other factors, such as diabetes balance, or the absence of comorbidities, such as retinopathy, which is the main predictor of MCI in our model.

Our study identified microalbuminuria as the third contributing factor to MCI. This parameter reflects underlying endothelial dysfunction, which is a critical pathway to both renal and cerebral microvascular disease [4]. This finding is consistent with research indicating an increased risk of cognitive disorders among patients with diabetic kidney disease. In a case-control study aimed at estimating the incidence and risk of neurocognitive disorders in patients with diabetic kidney disease compared to an age and sex-matched control cohort, the authors found that patients with diabetic kidney disease have a twofold increased risk of developing a neurocognitive disorder [22].

In our study, we found that serum LDL-cholesterol level is the fourth variable contributing to MCI. Our results suggest that individuals with lower LDL cholesterol levels have a protective effect against MCI, while higher levels are associated with an increased risk of developing MCI. Previous studies have shown that Low-density lipoprotein cholesterol (LDL-C) is a risk factor for MCI in patients with type 2 diabetes (OR: 1·635, P = 0·047) [23]. Indeed, LDL is involved in the genesis of atherosclerosis, including cerebral atherosclerosis, which leads to chronic cerebral ischemia and can contribute to the development of MCI. Chronic cerebral ischemia is recognized as a contributing factor to cognitive decline [4]. As LDL plays a crucial role in atherogenesis, its elevation in individuals with type 2 diabetes may exacerbate the risk of MCI by promoting atherosclerosis.

HbA1c levels were identified as the fifth contributing variable of MCI risk. Higher levels were linked to an increased risk, while not all lower levels were associated with a low risk. This pattern underscores the significant impact of glycemic control on cognitive function in patients with diabetes. Cukierman-Yaffe et al. demonstrated that high HbA1c levels are an independent predictor of lower cognitive function in individuals with Type 2 Diabetes [24]. However, it’s important to acknowledge that while elevated HbA1c levels are linked to cognitive decline, this association is not the strongest predictive factor in our study. However, it is important to acknowledge that while elevated HbA1c levels are linked to cognitive decline, this association is not the strongest predictive factor in our study. This ranking may reflect the complex interplay between hyperglycemia and hypoglycemia in cognitive outcomes. Higher HbA1c levels are associated with poor glycemic control, which in turn is linked to cognitive decline. However, hypoglycemia, a known factor for cognitive impairment [25], can lead to lower HbA1c levels, complicating the relationship between glycemic control and cognitive health.

The last parameter contributing to MCI in our study was serum creatinine level. Elevated levels, which indicate kidney dysfunction, were associated with an increased risk of MCI, while lower levels corresponded to a decreased risk. This pattern highlights the potential role of renal function as an indicator of MCI in patients with type 2 diabetes. An observational study using data from the National Health and Nutrition Examination Survey (NHANES) from 2011–2014 found that elevated serum creatinine was independently associated with lower scores on cognitive function tests in older diabetic patients [26]. Multiple mechanisms may contribute to this association, diabetes can cause microcirculatory dysfunction and inflammation, which can lead to hemodynamic disturbances that accelerate renal and cerebrovascular damage [22].

Finally, our study not only demonstrates the potential of machine learning for predicting mild cognitive impairment (MCI) among patients with type 2 diabetes but also identifies key predictive factors for this cognitive disorder. This model can help clinicians identify at-risk patients for MCI and allow for early detection in those with identified risk factors. Basic data handling skills and clinical knowledge are necessary for interpreting the model’s outputs effectively.

This study is the first to use machine learning models to predict MCI among patients with type 2 diabetes, to the best of our knowledge. The Extra Trees classifier accurately identifies crucial predictors of MCI. The limitations of our study include the relatively small sample size from a single hospital and the extensive use of variables. While this approach is beneficial for predictive accuracy, it could also lead to overfitting, despite our precautions. Additionally, medication data were not available in the dataset used, which may limit the interpretation of certain predictors. This limitation stems from the original cohort design, in which medication use was not documented.

## Conclusions

This study demonstrates the potential clinical application of machine learning models in identifying early MCI in patients with diabetes. Through the identification of key predictors, our study suggests avenues for early intervention that could slow the progression of MCI and prevent its evolution into Alzheimer’s disease. Our study emphasizes the importance of incorporating predictive analytics into clinical practice to enhance MCI management among patients with type 2 diabetes. However, additional larger studies are necessary to improve the accuracy and robustness of our model and enable the development of more precise predictors.

## Supporting information

S1 Fig Histograms of dataset variables to evaluate their distribution. Distribution plots for key variables used in the study.
(TIF)

S2 Fig Pearson correlation coefficients between the variables over vectors of all the samples. Heatmap showing correlation coefficients between clinical variables. BMI = body mass index; HBA1C=glycated hemoglobin;TG = triglyceride serum level; CT = cholesterol serum level;PAD = peripheral artery disease;CAD = coronary artery disease.
(TIF)

S1 Table Comparison of the performance of the predictive models using the training dataset after feature selection using recursive feature elimination. *Fine-tuned model using random grid search (sikit-learn library), ** Fine-tuned model using Optuna random search (Optuna library).
(PDF)
