# Supplementary figures and images for: Diabetic retinopathy as the primary predictor of mild cognitive impairment in type 2 diabetes: Insights from machine learning models
Source: PLoS One. 2025 Sep 26;20(9):e0332442. doi: 10.1371/journal.pone.0332442 (PMC12468980; doi:10.1371/journal.pone.0332442)

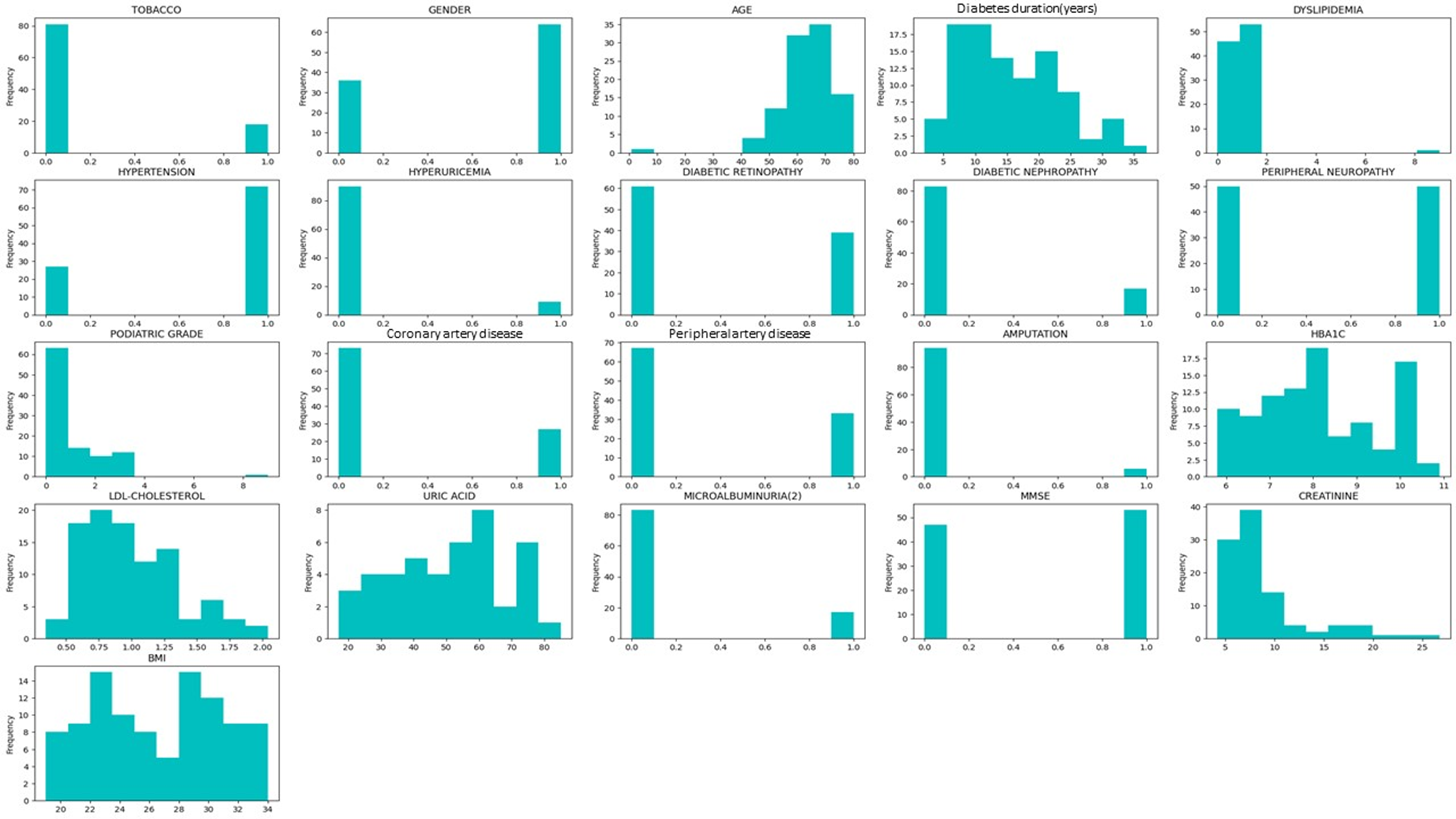

Supplement: S1 Fig — Distribution plots for key variables used in the study. (TIF) [file pone.0332442.s001.tif]

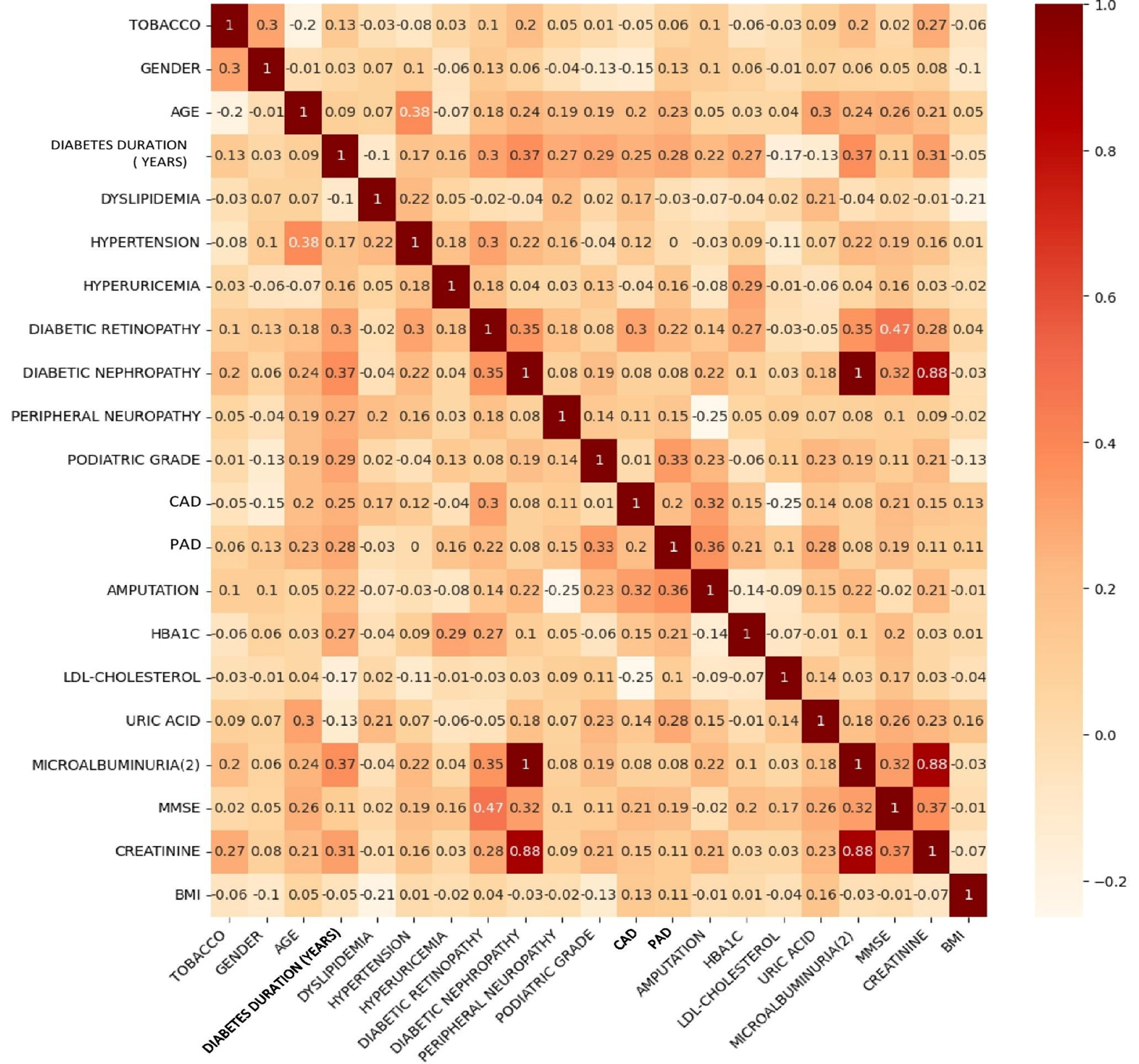

Supplement: S2 Fig — Heatmap showing correlation coefficients between clinical variables. BMI = body mass index; HBA1C=glycated hemoglobin;TG = triglyceride serum level; CT = cholesterol serum level;PAD = peripheral artery disease;CAD = coronary artery disease. (TIF) [file pone.0332442.s002.tif]
