# Supplementary material for: Diabetic retinopathy as the primary predictor of mild cognitive impairment in type 2 diabetes: Insights from machine learning models
Source: PLoS One. 2025 Sep 26;20(9):e0332442. doi: 10.1371/journal.pone.0332442 (PMC12468980; doi:10.1371/journal.pone.0332442)
Supplement: S1 Table — *Fine-tuned model using random grid search (sikit-learn library), ** Fine-tuned model using Optuna random search (Optuna library). (PDF) [file pone.0332442.s003.pdf]

S1 Table.Comparison of the performance of the predictive models using the training dataset after Features selection using Recursive Feature Elimination.

**Machine learning Model**	**Accuracy**	**AUC**	**Recall**	**Precision**	**F1**
Gradient Boosting Classifier*	0·8476	0·8083	0·8333	0·9000	0·8586
Decision Tree Classifier	0·8333	0·8292	0·8583	0·8483	0·8427
CatBoost Classifier*	0·8310	0·8528	0·8250	0·8633	0·8356
Extra Trees Classifier**	0·8024	0·8014	0·7333	0·8600	0·7768
Random Forest Classifier	0·8000	0·8583	0·7917	0·8433	0·8044
Logistic Regression	0·7548	0·7667	0·6833	0·8300	0·7346
Extreme Gradient Boosting**	0·7500	0·7806	0·8750	0·7167	0·7833
Light Gradient Boosting Machine	0·7429	0·7806	0·6583	0·8333	0·7314
Ridge Classifier	0·7405	0·0000	0·6583	0·8300	0·7079
Quadratic Discriminant Analysis	0·7405	0·6944	0·6500	0·8383	0·7172
Linear Discriminant Analysis	0·7405	0·7667	0·6583	0·8300	0·7079
Naive Bayes	0·7286	0·7194	0·5917	0·8500	0·6700
K Neighbors Classifier	0·6929	0·7764	0·6500	0·7550	0·6710
SVM - Linear Kernel	0·6333	0·0000	0·5583	0·6650	0·5812
Dummy Classifier	0·5286	0·5000	1·0000	0·5286	0·6903

*Fine-tuned model using random grid search (sikit-learn library), ** Fine-tuned model using Optuna random search (Optuna library)
